# The Role of Na:K:2Cl Cotransporter 1 (NKCC1/SLC12A2) in Dental Epithelium during Enamel Formation in Mice

**DOI:** 10.3389/fphys.2017.00924

**Published:** 2017-11-21

**Authors:** Rozita Jalali, Johannes C. Lodder, Behrouz Zandieh-Doulabi, Dimitra Micha, James E. Melvin, Marcelo A. Catalan, Huibert D. Mansvelder, Pamela DenBesten, Antonius Bronckers

**Affiliations:** ^1^Department of Oral Cell Biology, Academic Centre for Dentistry Amsterdam (ACTA), Amsterdam Movement Sciences, University of Amsterdam, VU University Amsterdam, Amsterdam, Netherlands; ^2^Department of Functional Anatomy, Academic Centre for Dentistry Amsterdam (ACTA), MOVE Research Institute Amsterdam, University of Amsterdam, VU University Amsterdam, Amsterdam, Netherlands; ^3^Department Integrative Neurophysiology, Center for Neurogenomics and Cognitive Research, VU University, Amsterdam, Netherlands; ^4^Department of Clinical Genetics, VU University Medical Center, Amsterdam Movement Sciences, Netherlands; ^5^Secretory Mechanisms and Dysfunction Section, NIDCR/NIH, Bethesda, MD, United States; ^6^Departamento de Ciencias Químicas y Farmaceúticas, Facultad de Ciencias de la Salud, Universidad Arturo Prat, Iquique, Chile; ^7^Department of Orofacial Sciences, School of Dentistry, University of California, San Francisco, San Francisco, CA, United States

**Keywords:** mineralization, ion transport, pH regulation, SLC26A, gap junctions

## Abstract

Na^+^:K^+^:2Cl^−^ cotransporters (NKCCs) belong to the *SLC12A* family of cation-coupled Cl^−^ transporters. We investigated whether enamel-producing mouse ameloblasts express NKCCs. Transcripts for *Nkcc1* were identified in the mouse dental epithelium by RT-qPCR and NKCC1 protein was immunolocalized in outer enamel epithelium and in the papillary layer but not the ameloblast layer. In incisors of *Nkcc1*-null mice late maturation ameloblasts were disorganized, shorter and the mineral density of the enamel was reduced by 10% compared to wild-type controls. Protein levels of gap junction protein connexin 43, Na^+^-dependent bicarbonate cotransporter e1 (NBCe1), and the Cl^−^-dependent bicarbonate exchangers SLC26A3 and SLC26A6 were *upregulated* in *Nkcc1*-null enamel organs while the level of NCKX4/SLC24A4, the major K^+^, Na^+^ dependent Ca^2+^ transporter in maturation ameloblasts, was slightly downregulated. Whole-cell voltage clamp studies on rat ameloblast-like HAT-7 cells indicated that bumetanide increased ion-channel activity conducting outward currents. Bumetanide also reduced cell volume of HAT-7 cells. We concluded that non-ameloblast dental epithelium expresses NKCC1 to regulate cell volume in enamel organ and provide ameloblasts with Na^+^, K^+^ and Cl^−^ ions required for the transport of mineral- and bicarbonate-ions into enamel. Absence of functional *Nkcc1* likely is compensated by other types of ion channels and ion transporters. The increased amount of Cx43 in enamel organ cells in *Nkcc1*-null mice suggests that these cells display a higher number of gap junctions to increase intercellular communication.

## Introduction

Ion transport by ameloblasts is critical for the formation of fully mineralized dental enamel. Disturbance in transport of mineral and/or bicarbonate ions (either local and/or systemic) during enamel development can lead to permanent enamel abnormalities (Lacruz et al., 2010a, 2012).

To form and mature apatite crystals, maturation ameloblasts transport mineral ions as calcium and phosphate into the forming enamel using apical plasma membrane transporter(s) such as Na^+^-Ca^2+^exchangers (NXC1,3) (Okumura et al., 2010) and Na^+^-K^+^-Ca^2+^ exchangers (NCKX4/SLC24A family) (Hu et al., 2012; Parry et al., 2013; Bronckers et al., 2015a). However, formation of each unit cell of the hydroxyapatite crystal also releases ~8 protons (Ryu et al., 1998) that need to be buffered to prevent acidification and arrest of mineral accretion. In order to buffer protons, ameloblasts have been proposed to secrete bicarbonate ions into the enamel space that neutralize H^+^ (Smith, 1998). Bicarbonate is taken up by ameloblasts basolaterally from the extracellular fluid by bicarbonate-transporting proteins such as electrogenic (NBCe) and electroneutral (NBCn) Na^+^-dependent bicarbonate cotransporters expressed in the ameloblasts during secretion- and maturation phase of amelogenesis (Josephsen et al., 2010; Lacruz et al., 2013; Jalali et al., 2014; Bori et al., 2016). During maturation phase ameloblasts also generate bicarbonates by producing carbonic acid (H_2_CO_3_) from CO_2_ and H_2_O via cytosolic carbonic anhydrase 2 (Car2) (Bori et al., 2016). During the maturation phase the bicarbonate ions are secreted by ameloblasts into the enamel space by coordinated activity of basolateral AE2, and apically by CFTR and members of the SLC26A family: SLC26A1, SLC26A3/Dra, SLC26A4/Pendrin, SLC26A6/Pat1 and SLC26A7 (Lyaruu et al., 2008; Lacruz et al., 2010b, 2012, 2013; Bronckers et al., 2011, 2015a; Jalali et al., 2015; Yin et al., 2015).

Recent studies suggested that non-ameloblast epithelial cells connected to the basal side of the ameloblasts are also involved in transepithelial transport, based on the expression of NBCe1 (Josephsen et al., 2010; Lacruz et al., 2010b; Jalali et al., 2014) and Na^+^-K^+^-ATPase and ATPase activity in papillary layer (Garant and Sasaki, 1986; Glynn, 2002; Josephsen et al., 2010). Accumulation of Na^+^ and K^+^ and reduction of Cl^−^ in hypomineralizing enamel of *Cftr*- null and *Ae2*- null mice suggested that in wild type mice Na^+^ and K^+^ are removed but that Cl^−^ is taken up from the forming maturation enamel (Bronckers et al., 2015a). It was speculated that maturation ameloblasts remove Na^+^ and K^+^ from the enamel space by a yet unknown mechanism which is impaired in *Cftr*-null and *Ae2*-null mice. Potential transporters for potassium and sodium reabsorption in ameloblasts may correspond to Na^+^:K^+^:2Cl^−^ (NKCC) cotransporters, which are sensitive to loop diuretics such as bumetanide and furosemide (Gagnon et al., 1998).

Na^+^-K^+^-Cl^−^ cotransporters (NKCCs) aid in the active transport of sodium, potassium and chloride in or out of the cells (Haas, 1994). The SLC12A family contains two Na^+^:K^+^:2Cl^−^ cotransporters, NKCC1 and NKCC2, encoded by two different genes (*SLC12A2* and *SLC12A1*, respectively). NKCC1 is widely distributed throughout the body. In salivary glands, basolateral NKCC1 mediates the transport of sodium, potassium and chloride from blood into the acinar cells. Lack of functional NKCC1 results in dramatic reduction of the volume of secreted saliva (Evans et al., 2000). NKCC1 is also necessary for establishing the potassium-rich endolymph that bathes part of the cochlea, the organ necessary for hearing. Inhibition of NKCC1, as with furosemide or bumetamide, can result in deafness (Delpire et al., 1999). NKCC2 has a more restricted distribution and is specifically found in the apical membrane of cells in the thick ascending limb of the loop of Henle and the macula densa in nephrons where it serves both in sodium absorption and tubuloglomerular feedback (Lytle et al., 1995). Expression of *Slc12a2/Nkcc1* messenger RNA was reported in mouse enamel epithelium in bud to bell stage teeth (embryonic stage E15–postnatal day 3), suggesting a possible involvement of NKCC1 in enamel organ development (Hübner et al., 2001).

In this study, we tested the hypothesis that NKCC1 plays a role in the ion transport by dental epithelium during enamel formation. The enamel organs of mice and HAT-7 cells, a rat ameloblast-like cell line derived from the cervical loop of a rat incisor (Kawano et al., 2002), were analyzed for expression of NKCC1 at the protein level. The effect of the null mutation of *Nkcc1* on enamel development, cell-size and enamel mineralization was studied by histology, immunohistochemistry, micro-computed tomography and Western blotting. To understand the role of NKCC1 cell volume regulation, we exposed *in vitro* HAT-7 cells to bumetanide and measured cell volume using the calcein-quenching method (Ye et al., 2015). The effect of bumetamide was also tested on electrophysiology of HAT-7 membranes by patch clamp.

## Materials and methods

### Tissues

*Nkcc1*-null mutant mice used for this study were generated and genotyped as previously described (Flagella et al., 1999). One hemi-maxillary incisor of each mouse was used for immunohistological studies and the other one was freeze-dried for micro-CT analysis and western blotting. For each genotypic mouse strain, at least three wild-type mice and three null mutant mice were analyzed.

All experiments were approved by the Committee for Animal Care (Vrije Universiteit Amsterdam; ACTA-12-01) and by the Animal Care and Use Committee of the National Institute of Dental and Craniofacial Research, National Institutes of Health (ASP 13–686). The methods were carried out in accordance with the approved guidelines.

### Cell culture

HAT-7 cells were grown in DMEM/F12 (Sigma-Aldrich, St. Louis, MO, USA) with 10% HyClone fetal bovine serum, 100 U/mL of penicillin, 10 μg/mL of streptomycin (Sigma-Aldrich) and 10^−5^ mM dexamethasone in humidified atmosphere containing 5% CO_2_ at 37°C (Bori et al., 2016).

### Histology

Mouse jaws were fixed by immersion in 5% paraformaldehyde in 0.1 M phosphate buffer pH 7.3 and embedded in paraffin. Calcified tissues from mice older than 2 weeks were first decalcified in 4% EDTA, pH 7.3 for 2–3 weeks at 4°C. Salivary glands were fixed in Bouin's fixative (75 ml of saturated picric acid, 25 ml of 40% formaldehyde, and 5 ml of glacial acetic acid) at room temperature for 48 h and processed into 5–7 μm thick paraffin sections. Dewaxed sections were stained with 1% hematoxylin (1 min) and eosin (5 min) (HE) or used for immunohistochemicals staining.

### Quantifications in cells of the enamel organ

At 40x objective pictures of secretory and maturation stage of enamel organ were made and images selected based on the anatomical position in the tooth. The length of the long axis of secretory and maturation ameloblasts was measured in sagittal sections using imaging software (image J).

### Real time quantitative polymerase chain reaction (RT-qPCR)

Total RNA was extracted from HAT-7 cells and various fresh mouse tissues using the NucleoSpin RNA/protein kit (Macherey-Nagel, Düren, Germany) according to the manufacturer's instructions. First strand cDNA synthesis was performed in a 20 μl reverse transcription reaction containing 200 ng of total RNA using VILO kit (Invitrogen) according to the manufacturer‘s instructions. Real-time PCR analysis was performed to analyze expression of *Slc12a2* (*Nkcc1*) with the primer sequences (FW:5' GAAGAAAGTACTCCAACCAGAGATG 3'; REV: 5' CTGAAGTAGACAATCCTGTGATA 3'; size: 232 bps) and the housekeeping protein tyrosine 3-monooxygenase (*Ywhaz*) with sequences (FW:5'GATGAAGCCATTGCTGAACTTG3'; REV:5'CTATTTGTGGGACAGCATGGA3'; size:229 bps) shown by using the LightCycler 480 system based on SYBR Green I dye (Roche Applied Science, Indianapolis, IN, USA). The LightCycler reactions were prepared in 20 μl total volume with 7 μl PCR-H_2_O, 0.5 μl forward primer (0.2 μM), 0.5 μl reverse primer (0.2 μM), 10 μl LightCycler Mastermix (LightCycler 480 SYBR Green I Master; Roche Applied Science, IN, USA), to which 2 μl of 5 times diluted cDNA was added as PCR template. Controls in the real-time RT-PCR reaction included RT reactions without the reverse transcriptase (control for DNA carry over) and RT reactions without template (control for reagent contamination). With the Light Cycler software, the crossing points were assessed based on a standard curve of five serial dilutions ranging from 10 ng to 1.6 pg of cDNA. PCR efficiency (E) was automatically calculated using the fit point method (*E* = 10^−1^/slope). Gene expression data were used only if the PCR efficiency was within a 1.85–2.0 range. For each gene the amount of measured DNA was normalized to that of YWHAZ housekeeping gene to calculate relative gene expression. The relative gene expression in different tissues was normalized to kidney levels for each gene in the graphs.

### Immunohistochemistry

Dewaxed paraffin sections were rinsed in phosphate buffered saline (PBS) and subjected to antigen retrieval in 10 mM citrate buffer (pH 6.0) either at 60°C overnight or for 20 min in microwave at 95°C. Endogenous peroxidase was blocked with a peroxidase block solution (Envision kit, Dakocytomation) for 5 min. Sections were washed 3x in tris-buffered saline (TBS). Non-specific staining was blocked for 30 min with 2% BSA after which sections were incubated overnight at 4°C with primary antibodies. These were (1) goat anti-NKCC1 (Santa Cruz, affinity purified, catalog number SC-21545), raised against the N-terminal end of human NKCC1. (2) Mouse anti-NCKX4 monoclonal antibodies (IgG2b isotype) from NeuroMab (UC Davis/NIH NeuroMab Facility, catalog # N414/25). (3) Matched non-immune IgG (1:200–1:300) or normal serum (same concentration as primary antibodies) served as controls. After overnight incubation at 4°C with primary antibodies, sections were washed three times in TBS and incubated with rabbit anti-goat secondary antibody conjugated to peroxidase (Thermo Scientific) for 1 h at room temperature. After washing staining was visualized using DAB (EnVision kit), counterstained with hematoxylin. For immunofluorescent staining, goat anti mouse–IgG conjugated to Alexa Fluor 488 (5 μg/mL; Invitrogen) was used and counterstained with propidium iodine (Vector Laboratories, Burlingame, CA, USA). Immunohistochemistry images were acquired with a Leica EL6000 or Axio Zoom V16 microscope.

### Microcomputed tomography (microCT)

To determine the degree of mineral content, hemi-maxillae were scanned at a resolution of 8 μm voxels in a microCT-40 high-resolution scanner (Scanco Medical, AG, Bassersdorf, Switzerland) to measure mineral density in enamel. An internal standard made of solid-sintered apatite (5-mm diameter, 1.5–2.0 mm thick, solid sintered) with density of 2.9 ± 0.2 g/mL (a gift from Himed; http://www.himed.com) was used as high-density standard. Beginning at the apical part of the incisor and moving toward the tip, cross-sectioned images through the incisors were collected at sequential intervals of 300 μm in maturation-stage and 60 μm in secretory-stage enamel. In each slice, the mineral density of enamel was measured halfway through the enamel layer at three sites within a circular area, with a diameter of 7 μm at the mesial, lateral, and central sides. Mean values and standard error of mean (SEM) of the mineral density were calculated and presented as mean ±SEM. Independent Student's *t*-test was used to compare the groups. Statistical significance was set at *p* < 0.05 level.

### Western blotting

From freeze-dried upper incisors obtained from wild-type and *Nkcc1*^−/−^ mice early maturation stage enamel organs were micro dissected. The apical half of the enamel organ was dissected, dissolved in non-reducing condition in SDS loading buffer (from Nucleospin Triprep kit, Macherey-Nagel, supplied by Bioke, Leiden, NL) and protein was measured using the BCA protein assay (Bio-Rad, Hercules, CA). Twenty micro gram of enamel organ denatured protein and 10 μg of molecular weight markers [Novex® Sharp Pre-stained Protein Standard (# LC5800) or SeeBlue® Plus2 Pre-stained Protein Standard (#LC5925)] were subjected to electrophoresis in a 3–8% Tris acetate Nupage gel with Tris acetate running buffer for 60 min at 150 V or 4–12% Bis-Tris Protein Gels with MOPS buffer for 35 min at 200 V. and subsequently electroblotted by an iBlot device (Invitrogen) on nitrocellulose membrane according to the manufacturer's instructions. Membranes were blocked with BSA 2% for 1 h at room temperature and incubated overnight (4°C) with the primary antibodies. Blots were washed three times in PBS and incubated with IRDye secondary antibodies (LI-COR). Visualization and quantification was carried out with the LI-COR Odyssey scanner and software (LI-COR Biosciences). Actin was detected at 680 nm wavelength (shown as red) and other primary antibodies and tubulin were detected at 800 nm wavelength (shown as green). Quantification was performed using Odyssey software. Intensity values of the bands were normalized for actin or tubulin and expressed as percentage of wild type (100%). For western blots the following primary antibodies were used: rabbit anti-SLC26A3/Dra (Research Genetics, Huntsville, AL, USA) (Jalali et al., 2015), rabbit anti-SLC26A6/Pat1 (donated by Dr. P. Aronson, Yale University, New Haven, CT, USA) (Jalali et al., 2015), rabbit anti-NBCe1 (Jalali et al., 2014) (donated by Dr. W.F. Boron, Case Western Reserve University, Cleveland, Ohio, USA), rabbit anti-NCKX4 (NeuroMab, UC Davis/NIH, # N414/25), rabbit anti-connexin (Abcam, #ab11370), mouse anti-β-actin antibody (Sigma, A2228) and rabbit anti-tubulin antibody (Abcam, ab59680). Secondary antibodies: IRDye 800CW conjugated goat anti-rabbit IgG (H+L) highly cross-adsorbed (LI-COR; Product number: 926–32,211) and IRDye 680CW conjugated goat anti-mouse IgG (H+L) highly-cross adsorbed (LI-COR; Product number: 926–32,220). Dilutions: anti-β-actin and anti-tubulin (1:1,000); other primary antibodies (1:250); secondary antibodies (1:10,000).

### Imaging of volume decrease after exposure to bumetanide

HAT-7 cells were loaded with 5 μM Calcein-AM (Molecular Probes) for 20 min at 37°C. Changes in cell volume of single HAT-7 cells, plated on poly-lysine coated glass cover slips, were assessed by measuring calcein fluorescence using the calcein-quenching method (Ye et al., 2015). Cells were bathed in iso-osmotic solution (20–22°C) and transferred to a continuously perfused (5 ml/min) recording chamber, equipped with a microscope with 10X objective. An image was taken every 30 s. At the start images were obtained for 5 min in the iso-osmotic solution to establish the baseline. Cells were exposed to media supplemented with 10 μM bumetanide for 30 min, after which the iso-osmotic solution was re-introduced and images were taken for another 10 min. The cell surface and average fluorescence of each cell in the acquired images was calculated using Image-J software. Changes in cell surface and average fluorescence were expressed as S_t_/S_0_ and F_t_/F_0_ ratios, respectively, where S_0_ and F_0_ are the average cell surface area and fluorescence under iso-osmotic treatment at the beginning of the experiment.

### Electrophysiological recordings

Cover slips bearing HAT-7 cells were transferred to the recording chamber, containing 1.5 ml external solution. The external solution was changed at a rate of 1.5 ml/min using a gravity driven constant perfusion system. During the recordings, HAT-7 cells were perfused with standard artificial Cerebral Spinal Fluid (aCSF) containing (in mM): 126 NaCl, 3KCl, 10 D-glucose, 26 NaHCO_3_, 1.2 NaH_2_PO_4_, 2 CaCl_2_ and 1 MgSO_4_, carboxygenated with 95% O_2_ and 5% CO_2_ to obtain pH 7.4 and an osmolality of 300 mOsm. For electrophysiological recordings, we used a EPC-8 amplifier and a Instrutech ICT-18 (all from Heka, D-67466 Lambrecht, Germany). Cells were identified and patched using an Olympus IX51 inverted microscope equipped with a LCAcn 40x 0.55nA ph2 objective (all Olympus corporation, Tokyo, Japan). Glass pipettes for whole-cell and cell attached recordings were made from borosilicate capillaries (OD 1.5 mm, ID 0.86 mm; Harvard Apparatus, Holliston, MA, USA) using a Sutter P-87 micro-electrode puller (Sutter instruments, USA) and displayed a resistance of 2.5–5 MΩ. Glass microelectrodes were filled with intracellular solution containing (in mM): 110 K-Gluconate, 10 KCl, 10 HEPES, 0.4 NaGTP, 4 Mg_2_ATP and 10 K-Phosphocreatine (pH 7.3 adjusted with KOH, 290 mOsm). HAT-7 cells were gently lifted from the cover slip and placed in front of a piezo-driven theta-barrel electrode (TGC 200; Harvard Apparatus, Holliston, MA, USA), filled with standard aCSF on one side and standard aCSF supplemented with 10 μM bumetanide (B1158000 Sigma-Aldrich) on the other side. By changing the position of the barrel bumetanide was applied during the whole-cell recording. Voltage ramps from −70 to 80 mV (500 ms) were applied under control and in the presence of 10 μM bumetanide.

The cell attached recordings were made by filling the recording pipet with aCSF for the control recordings and with aCSF supplemented 10 μM bumetanide for experimental recordings. All data was acquired using an internal 7-pole Bessel filter (5 kHz) and a sample frequency of 20 kHz. Recordings with an access resistance above 12 mΩ were excluded form analysis.

## Results

### *Slc12a2* mRNA expression in mouse tissues and rat HAT-7 cells

**Figure 1:**
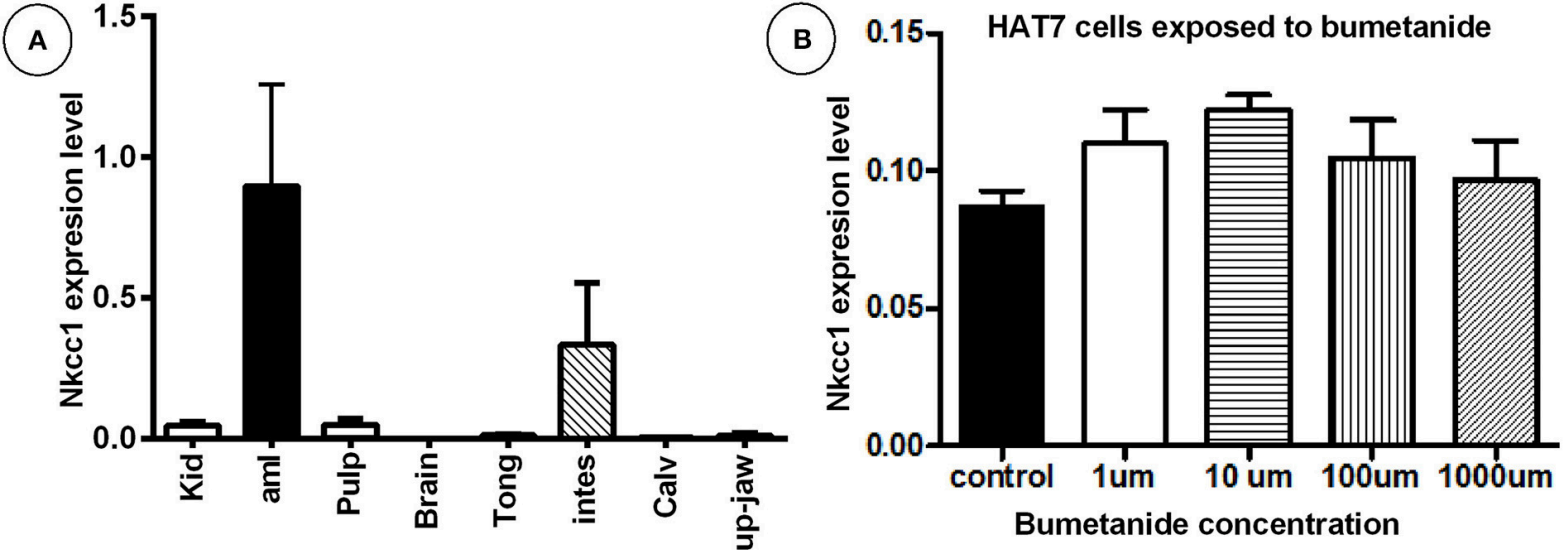
High mRNA expression of *Nkcc1* in mouse enamel organ **(A)** and HAT-7 cells **(B)** and effect of bumetanide on *Nkcc1* expression in HAT-7 cells **(B)**. In **(A)** total RNA was extracted from different tissues and *Nkcc1* expression values normalized for ywhaz. Tissues are listed along X-axis (average for *n* = 3 mice). **(B)** Total RNA was isolated from HAT-7 cells treated with zero (control) and different concentrations of bumetanide (1, 10, 100, and 1,000 μM). kid: kidney; amlb, ameloblasts/enamel organ; pulpa, pulp; tong: tongue; stom, stomac; m3calv, MC3T3 mouse calvarial cell line; intes, intestine; calv, calvaria.

Transcripts for *Nkcc1/Slc12a2* normalized for *Ywhaz* housekeeping gene were detectable in enamel organ and intestine (high), pulp and kidney moderate-(low); in the remaining tissues tested expression was very low or below detection limit (Figure 1A; Supplementary Figure 1). HAT-7 cells also expressed *Nkcc1* transcripts (Figure 1B).

Bumetanide blocks activity of the NKCC's. To test whether this blocking agent also could affect *Nkcc1* expression level in enamel epithelium, HAT-7 cells were exposed to various concentrations of this inhibitor for 45 min, washed, and incubated for 9 h in medium without inhibitor. Then total RNA was isolated and the amount of *Nkcc1* transcripts measured by RT-qPCR (Figure 1B). Bumetanide did not change the number of transcripts of *Nkcc1*.

### NKCC1 expression in HAT-7 cells, mouse enamel organ, and salivary glands

**Figure 2:**
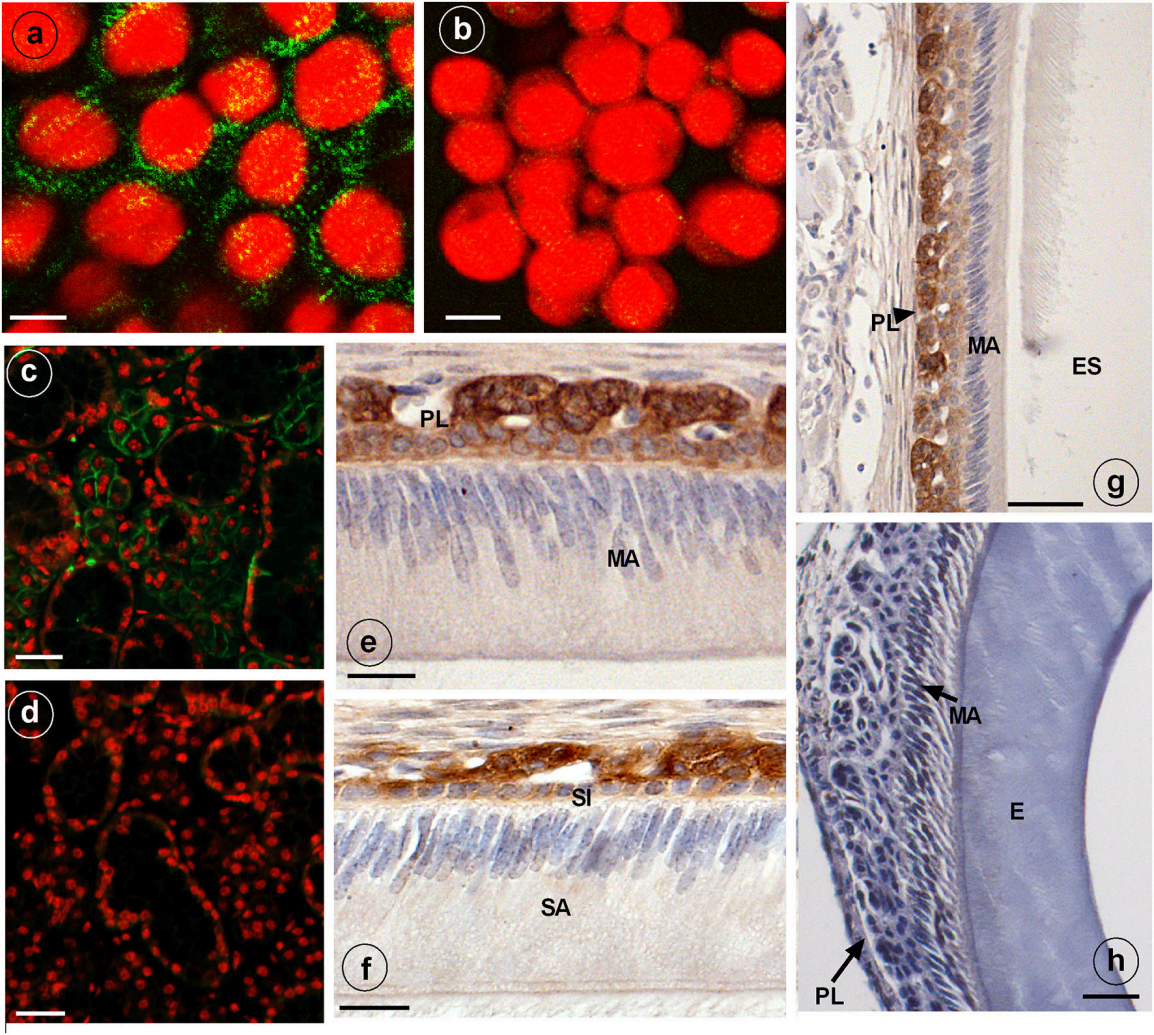
Immunostaining of NKCC1 protein in HAT-7 cells, developing mouse dental epithelium and mouse salivary glands. NKCC1 expression as green punctuate grains near the plasma membranes of cultured HAT-7 cells stained with anti-NKCC1 **(A)** or with non-immune IgG (**B**, control) and visualized using Alexa488-coupled secondary antibody. Panels **(C,D)** show salivary glands of a wild type **(C)** and *Nkcc1* null mouse **(G)** stained with anti-NKCC1. NKCC1 staining in mouse dental e*p*ithelium at secretory stage **(F)** and in maturation stage **(E,G)** in papillary layer. Absence of staining with anti-NKCC1 in salivary gland **(D)** and incisor **(H)** of a *Nkcc1*-null mouse confirms the specificity of primary antibody. Note: all the stainings have been tested in triplicate in three mice. E, enamel; ES, enamel space; SA, ameloblasts secretory stage; MA, ameloblasts maturation stage; P, pulp; PL, papillary layer; SI, stratum intermedium.

Anti-NKCC1 antibodies stained plasma membranes in HAT-7 cells as fine granular material (Figure 2A). Replacing primary (mouse) antibodies for normal non-immune mouse IgG failed to stain these membranes in HAT-7 cells (Figure 2B).

Strong immunostaining was detected in the basolateral plasma membranes of the acinar cells of salivary glands, a well-established site of NKCC1 expression (Evans et al., 2000) (Figure 2C).

In upper incisors, in presecretory stage and during the secretory and maturation stage, the plasma membrane of outer enamel epithelium cells was immunopositive for NKCC1 (Figures 2E,F; Supplementary Figure 2). No staining was seen in ameloblasts. Weaker staining was seen in dental epithelium between ameloblasts and outer enamel epithelium. Strong staining was apparent in the papillary layer (intracellular and membranes) during maturation (Figures 2E,G).

Sections from salivary glands (Figure 2D) and enamel organ from *Nkcc1*-null mice (Figure 2H) incubated with anti-NKCC1 failed to immunostain the tissues, validating the specificity of the antibodies. In developing molar tooth germs similar stainings were obtained: a strong staining for NKCC1 in outer enamel but no staining in ameloblasts (Supplementary Figure 3).

### NKCC1 expression is important for enamel organ function and enamel mineralization

**Figure 3:**
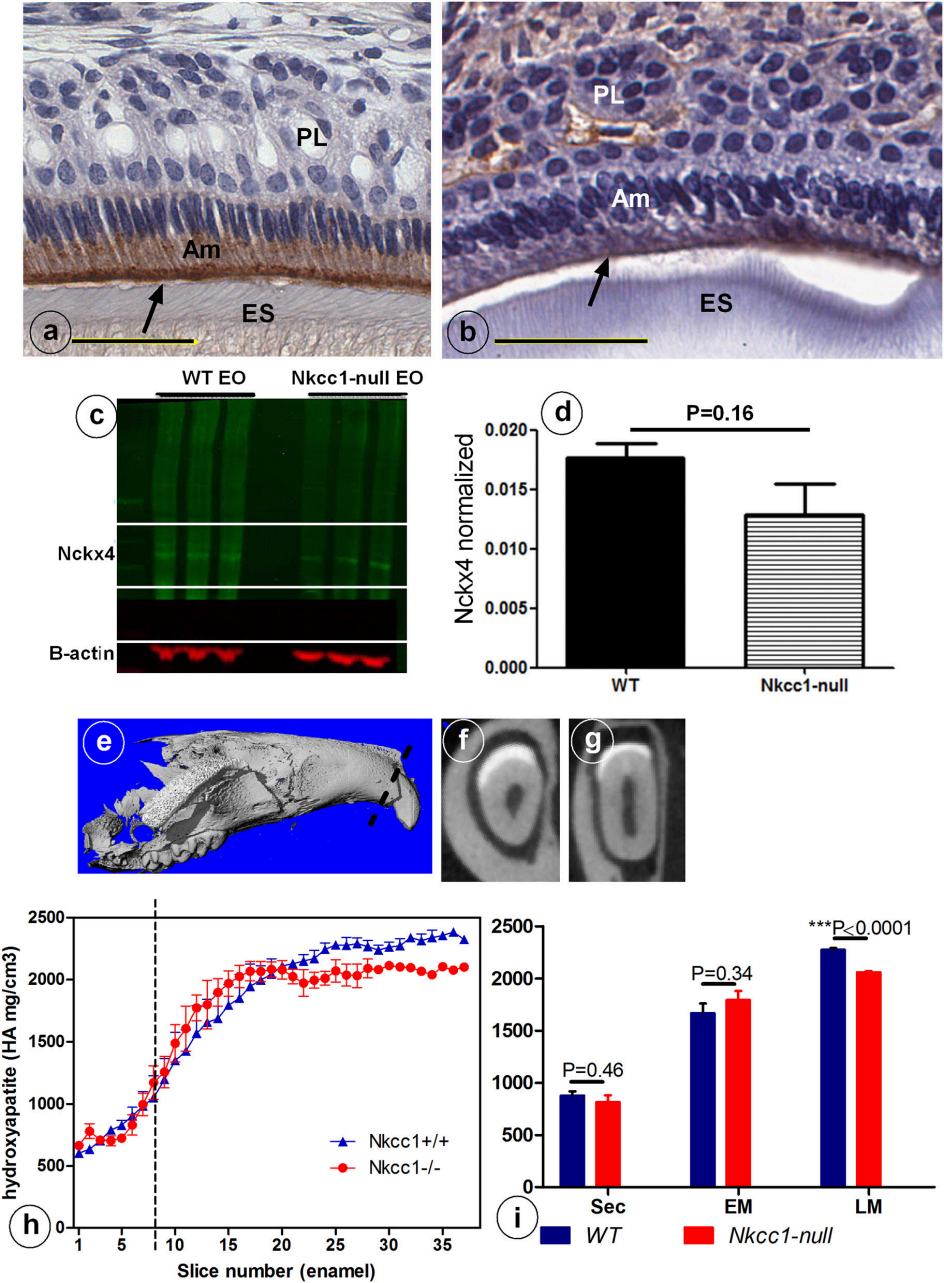
Reduced NCKX4 protein expression in enamel organ **(A–D)** and lower mineral density **(E–I)** of advanced maturation stage maxillary incisor enamel of *Nkcc1* null mice upper incisors (*n* = 3) **(A–D)** and decrease in maturation mineral density in mouse upper incisor (*n* = 3). Panels **(E–I)** shows the importance of NKCC1 for enamel mineralization. Nckx4 expression (arrows) in WT **(A)** and *Nkcc1*-null maturation ameloblast **(B)**. Total protein was extracted from WT and *Nkcc1*-null enamel organs (*n* = 3 mice) and NCKX4 expression analyzed by western blot **(C)**. Graph bar shows semi-quantitative NCKX4 band density normalized to that of ß-actin **(D)**. Mineral density measured by micro-CT plotted against slice numbers **(H)**. Blue color represents WT and red *Nkcc1*-null upper incisor; maturation stage starts at the dotted line. In **(I)** the bar graphs with the same color (blue and red) represent measurements of mineral density in different stages of amelogenesis (sec, secretory; EM, early maturation; LM, late maturation). Panels **(E–G)** show 3D reconstruction, virtual cross section of WT and *Nkcc1*-null upper incisor respectively. Circles in **(F)** indicate sites of measurement per slice.

*Slc12a2* (or *Nkcc1*) knockout mice exhibit growth retardation with a 30% incidence of death by the time of weaning. They are deaf, have less body fat, reduced mean arterial blood pressure, exhibit reduced cAMP-induced short circuit currents in jejunum, cecum, and trachea, unusual head postures, and engage in circling behaviors and rapid spinning, but have difficulty maintaining their balance (Flagella et al., 1999). Incisors of *Nkcc1*-null mice showed no obvious gross changes. Analysis of upper incisor enamel of *Nkcc1*-null mice showed no significant change in mineral density in enamel during secretory stage but a reduction at maturation stage, reaching a final density that was reduced about 10% compared to controls (*P* < 0.0001) (Figures 3E–I).

Next we examined expression of NCKX4 (sodium/calcium-potassium exchanger-4) a major transcellular calcium transporter in maturation ameloblasts, to determine whether calcium transport is affected in *Nkcc1*-null incisors. Nckx4 protein was strongly expressed in the apical membrane of maturation ameloblasts of wild type mice (Figure 3A), but its antibody-associated staining was weaker in *Nkcc1*-null mice (Figure 3B). Western blot analysis (Figure 3C) of protein extracts from wild type and *Nkcc1*-null maturation enamel organ, showed a small (but not significant) reduction of NCKX4 protein in *Nkcc1*-null enamel organ (Figure 3D).

### Upregulation of SLC26A6, DRA, NBCe1 and gap junction (Cx43) protein expression in *Nkcc1*-null enamel organ by western blotting

**Figure 4:**
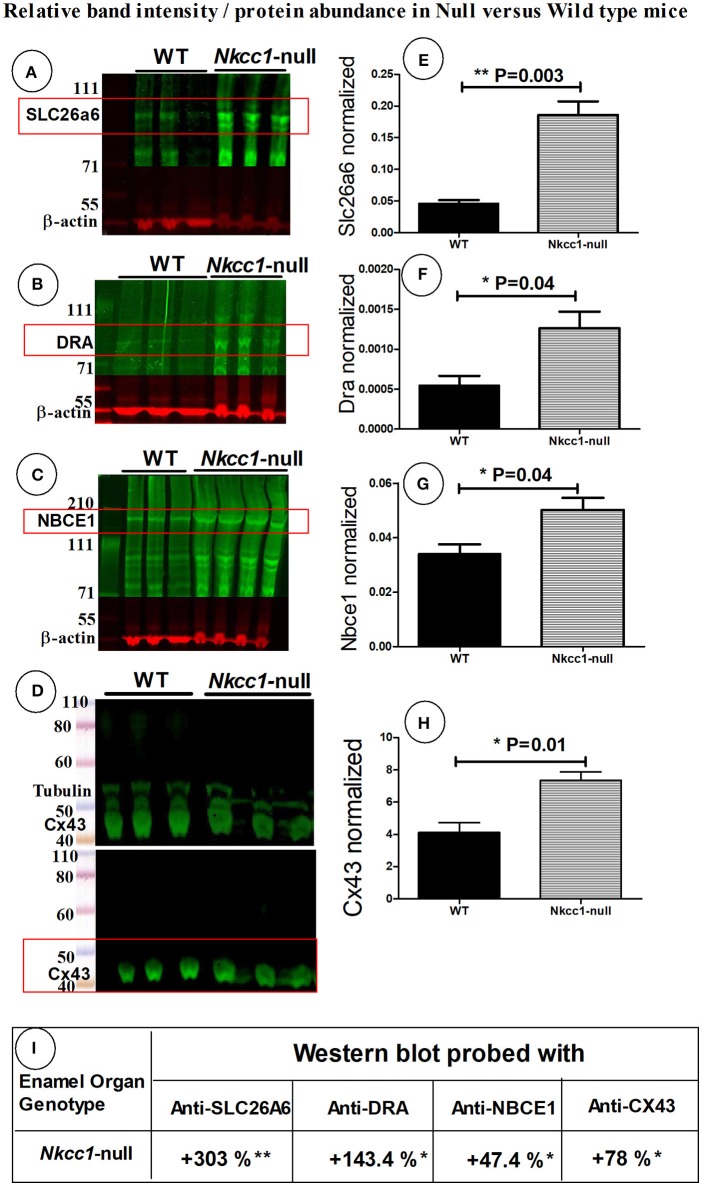
SLC26a6, DRA, NBCe1 and CX43 proteins possibly compensate during lost *Nkcc1* gene expression. Expression of SLC26a6 **(A)**, DRA **(B)**, NBCe1 **(C)**, and Cx43 **(D)** protein extracts of enamel organs of WT and *Nkcc1* null mice by western blotting. Graph bars **(E–G)** show semi-quantitative SLC26a6, DRA, NBCe1 bands normalized to ß-actin. The CX43 bands were normalized to tubulin **(H)**. Table **(I)** presents a summary of the quantification.

To examine if the expression of other Na^+^ and Cl^−^ ion transporters or/ and gap junction channels could compensate for the absence of NKCC1, we tested maturation stage enamel organs of *Nkcc1*-null mice for changes in protein levels of SLC26A6, DRA, NBCe1 and Cx43 by Western blotting (Figures 4A–H). Total protein extracted from *Nkcc1*-null enamel organs showed a 303% increase of SLC26A6/PAT1 (*P* = 0.003) (Figures 4A,E) compared with wild type a, 143% increase for SLC26A3/DRA (*P* = 0.04) (Figures 4B,F), 47% increase for NBCe1 (*P* = 0.04) (Figures 4C,G) and 78% increase for Cx43 (*P* = 0.01) (Figures 4D,H). The results are summarized in Figure 4I.

### Regulation of cell volume is impaired in *Nkcc1*-null enamel organ and HAT-7 cells exposed to bumetanide

**Figure 5:**
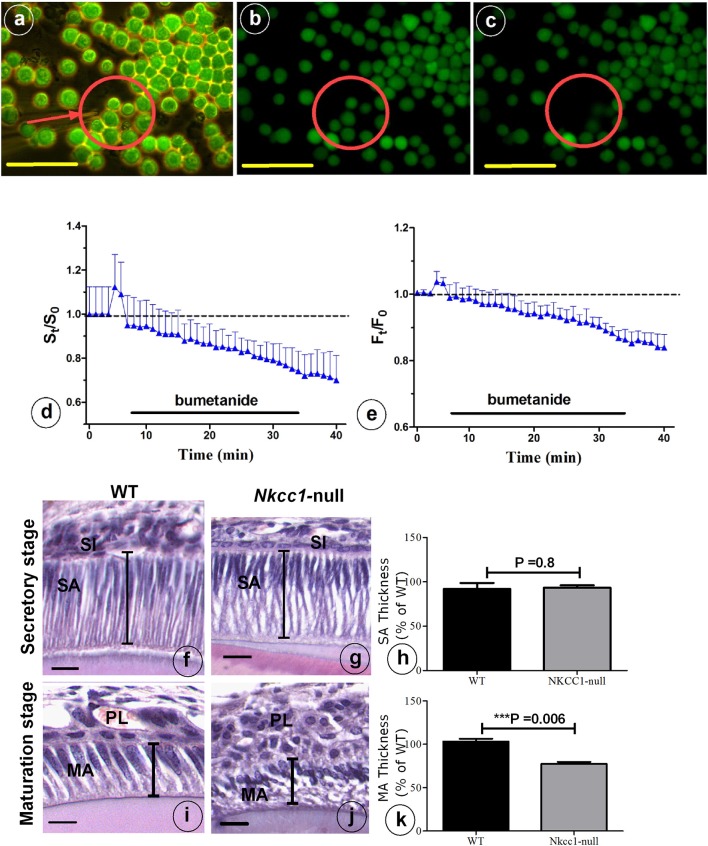
NKCC1-dependent regulatory volume decrease. **(A)** HAT-7 cells were loaded with 5 μM Calcein-AM for 20 min. Cells were bathed in a iso-osmotic solution that was switched to 10 μM bumetanide solution for 30 min as indicated by black bars in **(D,E)**. Changes in cell volume of single HAT-7 cells (arrow in **A**). **(B)** 2 min after bumetanide exposure; **(C)** 7 min after bumetanide exposure. **(D)** Average relative cell surface and **(E)** fluorescence, obtained under iso-osmotic with and without 10 μM bumetanide (*n* = 10). Length of ameloblast cells in *Nkcc1*-null **(G–J)** and WT mice stained with hematoxylen-eosin was measured using Image-J software (*n* = 3). Bar diagrams show the difference between ameloblasts length in the secretion stage **(H)** and maturation stage **(K)** of amelogenesis in WT and *Nkcc1*-null mice. SA, secretory ameloblasts; MA, advanced maturation ameloblasts; SI, stratum intermedium; PL, papillary layer. Lines indicate length of the cells in **(F,G,I,J)**; results for **(F,G)** summarized in **(H)**, while results for **(I,J)** are summarized in **(K)**. Bars = 50 μm.

NKCC1 is one of the proteins involved in cell volume recovery after cell shrinkage in kidney (Walker et al., 2002). Analysis of histological sections of *Nkcc1*-null upper mouse incisors showed that late maturation ameloblasts lost their polarization and that the cells of the papillary layer were shorter compared to that seen in wild type controls. However, secretory stage ameloblasts and stratum intermedium seemed hardly or not affected in the null mutants in comparison to wild type mice (Figures 5F,G,I,J). To examine a possible role of NKCC1 in regulating cell volume in ameloblasts, we measured cell volume changes of single HAT-7 cells *in vitro* by measuring cell size (surface area measurements) and by using the calcein-quenching method before and after bumetanide (10 uM) treatment, (Figures 5A–E). Bumetanide decreased cell volume as measured directly (Figure 5D) and by loss of fluorescence (Figure 5E). In incisors of *Nkcc1*-null mice the values of the long axes of secretory ameloblasts were not different from those in wild types (Figures 5F–H) but were significantly shorter (~25%; *P* < 0.0001; Figures 5I–K) in maturation ameloblasts.

### Bumetanide increases membrane conductance in HAT-7 cells

**Figure 6:**
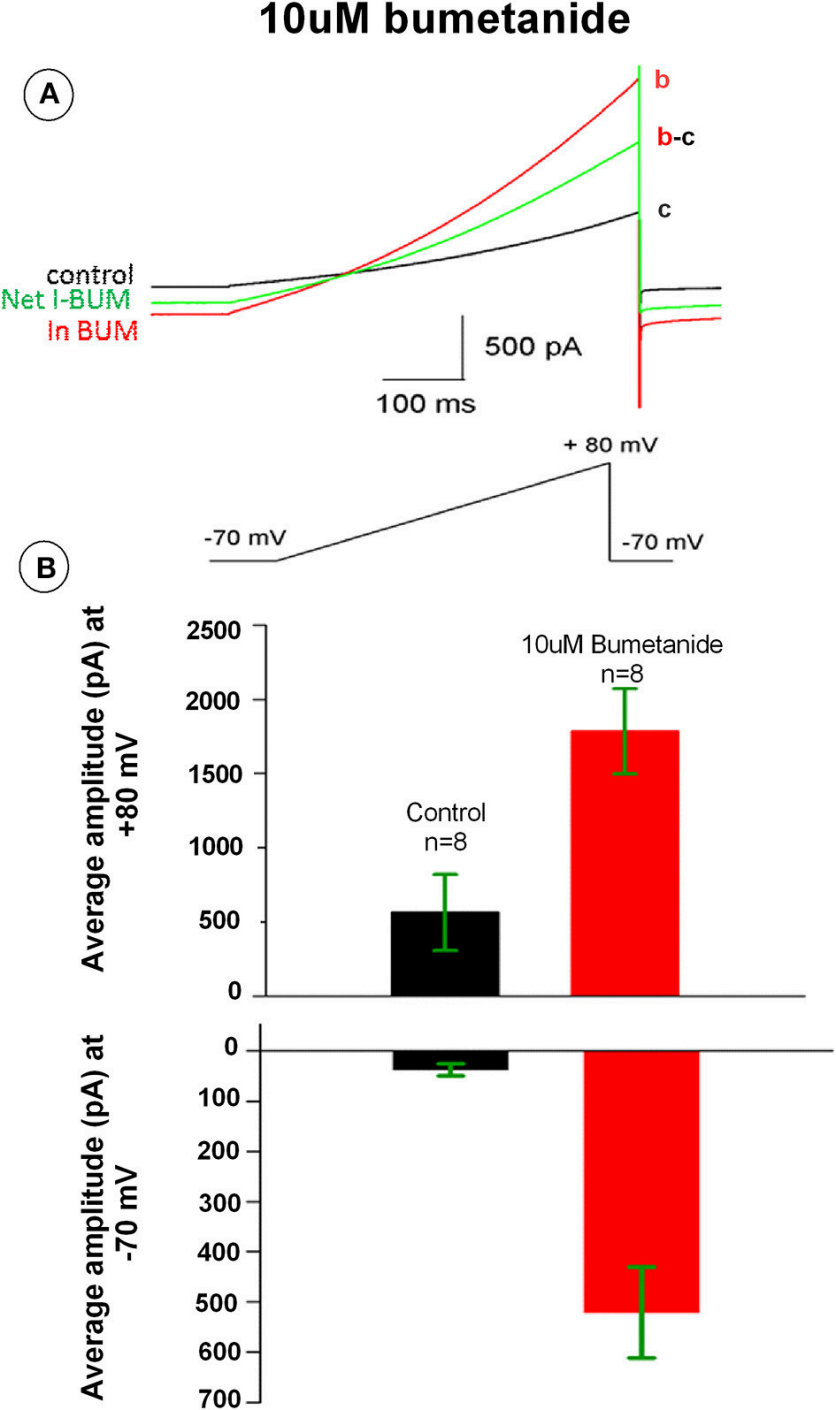
**(A)** Bumetanide 10 μM increased whole-cell membrane currents across the voltage range from −70 to +80 mV. The black tracing curve c is the control and the red aCSF tracing curve b is the current in the presence of 10 μM bumetanide. The green tracing curve b-c is the net current induced by 10 μM bumetanide. **(B)** Outward currents measured at +80 mV (upper) and inward current at −70 mV (lower). Bars show the average amplitude of control and 10 μM bumetanide (*n* = 8).

Bumetanide has been previously shown to induce potassium currents in canine kidney cells. To test whether bumetanide affects the membrane ionic conductance in HAT-7 cells, electrophysiological whole-cell voltage-clamp recordings were made from these cells. The membrane potential was ramped from −70 to +80 mV. In control conditions, the voltage ramp induced a small membrane current that reversed at −30 mV. Application of bumetanide (10 μM) increased membrane currents across the entire membrane potential range (Figure 6A). The outward current measured at +80 mV increased about 3-fold, whereas the inward current measured at −70 mV was 20-fold enlarged (Figures 6A,B). The increase of membrane currents induced by bumetanide hardly reversed upon washout (not shown). To our surprise, the bumetanide-induced currents reversed at −30 mV. Given that for the recording solutions used in these experiments the equilibrium potential for potassium was −95 mV, for chloride −65 mV and for sodium and calcium above +60 mV, this suggests that most likely a mixed ionic current was activated by bumetanide-block of NKCC transporters.

### Bumetanide increases ion channel activity in HAT-7 cells

**Figure 7:**
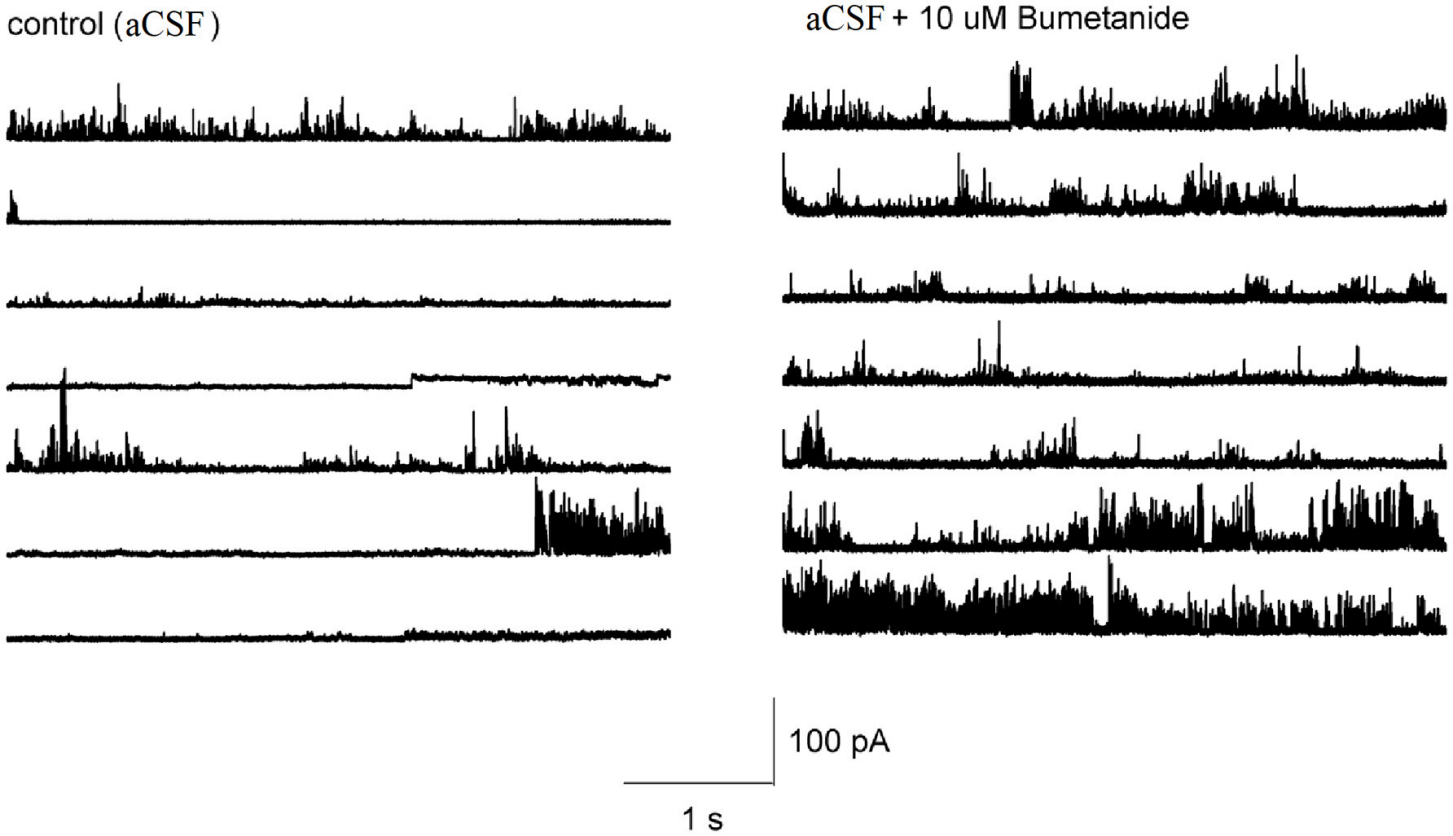
Representative tracings showing the effect of 10 μM bumetanide on the channel activity in HAT-7 cells. Left figure shows the channel activity obtained from cell-attached patch with aCSF in the recording pipet at 0 mV. Right figure shows the channel activity obtained when the recording pipet was filled with aCSF + 10 μM bumetanide and clamped at 0 mV.

It is not known whether the effect of bumetanide on the membrane conductance required block of NKCC transporters across the entire cell membrane, or whether this effect could also be induced locally. To determine this, we made cell-attached recordings from HAT-7 cells and applied bumetanide only to the membrane patch inside the patch pipette. The pipette potential was kept at 0 mV. In control recordings, ion channel activity and occasional discrete single channel openings were observed that were alternated by silent periods (*n* = 10, Figure 7). At this pipette potential, only outward currents were observed. During recordings in which bumetanide (10 uM) was included in the recording pipette, ion channel activity conducting outward currents increased (*n* = 10, Figure 7). No discrete amplitude levels could be distinguished, which suggests that blocking NKCC transporters with bumetanide augments the activity of multiple ion channels in the membrane patch. These results suggest that the effects of bumetanide on membrane ionic conductance could be induced locally in a patch of the cell membrane.

## Discussion

The present study demonstrates that in mice enamel organ epithelium in secretory and maturation stage expresses NKCC1 at the mRNA and protein level. Without NKCC1 enamel was slightly less mineralized, at visual inspection without gross change in enamel structure. The upregulation of NBCe1, DRA and PAT1 in enamel organs of *Nkcc1* null mice suggest that NKCC1 can be at least in part compensated by typical pH regulators as reported for salivary glands and duodenum epithelium in *Nkcc1* null mice (Evans et al., 2000; Walker et al., 2002). This suggests that NKCC1 is involved in enamel mineralization possibly by providing amloblasts Cl^−^ crucial for bicarbonate secretion but its role is limited. However, it cannot be excluded that some of the defects in enamel mineralization are secondary due to systemic changes brought about by changes in other tissues in *Nckk1*- null mice.

The reduction in mineral density of enamel in *Nkcc1* null mice was relatively small (10%) in comparison with the reduction in *Cftr*-null mice (47%, Bronckers et al., 2015b) and in *Ae2*- null mice (57% Lyaruu et al., 2014), two transporters critical for pH regulation by maturation ameloblasts. The reduction in mineral density in *Nkcc1* null mice could be due to: (1) a slightly increased acidification associated with reduced intracellular Cl^−^ levels in ameloblasts resulting from less NKCC1-mediated Cl^−^ transport in the papillary layer. The rather normal gross appearance of incisor enamel and the fact the mineral density changes appear quite late in comparison to the severe mineral reduction and porotic changes in enamel of *Cftr*-null and *Ae2*- null mice seen early in maturation make this option less likely. (2) Reduction of the cytosolic levels of K^+^ or a reduced number of apical NCKX4 in maturation ameloblasts needed for NCKX4-mediated Ca^2+^ transport by maturation ameloblasts. (3). Reduction in the levels of Na^+^ in ameloblasts required for Na-Pi2b mediated phosphate transport (Bronckers et al., 2015a).

Ameloblast did not express NKCC1 but papillary layer cells highly expressed the cotransporter. To reach forming enamel ions transported by NKCC1 into the papillary layer need to pass from papillary layer into ameloblasts. The enhanced expression of connex43 points to a mechanism for such transport. Ameloblasts and papillary layer have been suggested to act as a functional unit in passing ions from outer enamel epithelium to ameloblasts through gap junctions (Josephsen et al., 2010). Gap junctions are structures passing through the plasma membranes of two adjacent cells forming small pores that enable direct intracellular transport of ions and small molecules/peptides (Toth et al., 2010). The significance of communication between enamel organ cells is exemplified in Gja1Jrt^−/+^ mice in which enamel formation was severely affected by a decrease in the number of gap junctions in the enamel organ (Toth et al., 2010). Upregulation of connexin43 in enamel organ of *Nkcc1*-null mice may occur to increase the number of gap junctions to compensate for changes in ion transport due to absence of NKCC1.

Our histological and electrophysiological results suggest that NKCC1 is involved in amelogenesis. The distribution of NKCC1 in enamel organs, and the reduction in cell height of ameloblast layer in *Nkcc1*-null mice suggests that during amelogenesis the symporter NKCC1 serves to import Na^+^, K^+^, and Cl^−^ from blood vessels, regulates cell volume of dental epithelium and likely water transport across the ameloblast layer. Transport of large amounts of Cl^−^ and Na^+^ across epithelial cells, for example by Cl^−^ channels and cotransporters such as CFTR, NKCC2 and NKCC1, is required for fluid transport (e.g., in kidney) and maintenance or changes in cell volume. Volume changes in ameloblasts during normal amelogenesis are substantial indicated by the changes in the long axes of ameloblasts when moving from differentiation into secretion stage, reaching maximal values at full secretion stage, followed by a 50% reduction during transitional and early maturation stage. In *Nkcc1*-null mice the maturation ameloblasts were shorter than in wild type controls. Exposing HAT-7 cells to 10 μM bumetanide caused cell volume decrease without altering the number of *Nkcc1*-mRNA transcripts suggesting that iso-osmotically shrinkage accrued after adding bumetanide. These data are in line with a function of NKCC1 in cell volume regulation and ion transport.

In short, NKCC1 is highly expressed in non-ameloblast enamel organ epithelium. A reduction in mineral density of enamel, shortening of ameloblast cell body and upregulation of other ion transporters when NKCC1 is absent suggests that NKCC1 regulates cell volume and ion transport but can be partly compensated by enhanced activity of other ion transporters.

## Author contributions

Conceived and designed the experiments: RJ, AB, PD, and JL; Performed the experiments: RJ, JL, BZ-D, DM, JM, and MC; Wrote the manuscript: RJ, AB; Edited the manuscript: JL, DM, JM, MC, HM, PD, and AB.

### Conflict of interest statement

The authors declare that the research was conducted in the absence of any commercial or financial relationships that could be construed as a potential conflict of interest. The reviewer CC and handling Editor declared their shared affiliation.
