# Supplementary figures and images for: The Role of Na:K:2Cl Cotransporter 1 (NKCC1/SLC12A2) in Dental Epithelium during Enamel Formation in Mice
Source: Front Physiol. 2017 Nov 21;8:924. doi: 10.3389/fphys.2017.00924 (PMC5702478; doi:10.3389/fphys.2017.00924)

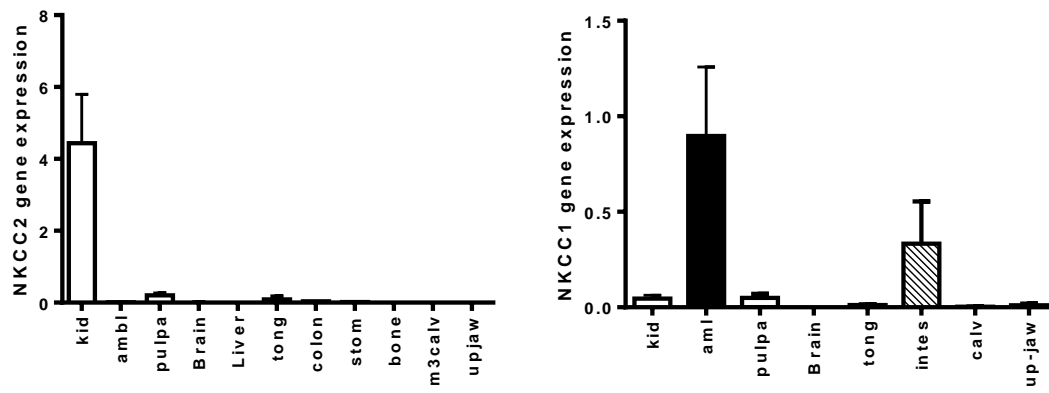

Supplement fig1. NKcc2 and Nkcc1 gene expression

Supplement: Supplementary file 1 [file DataSheet1.PDF]

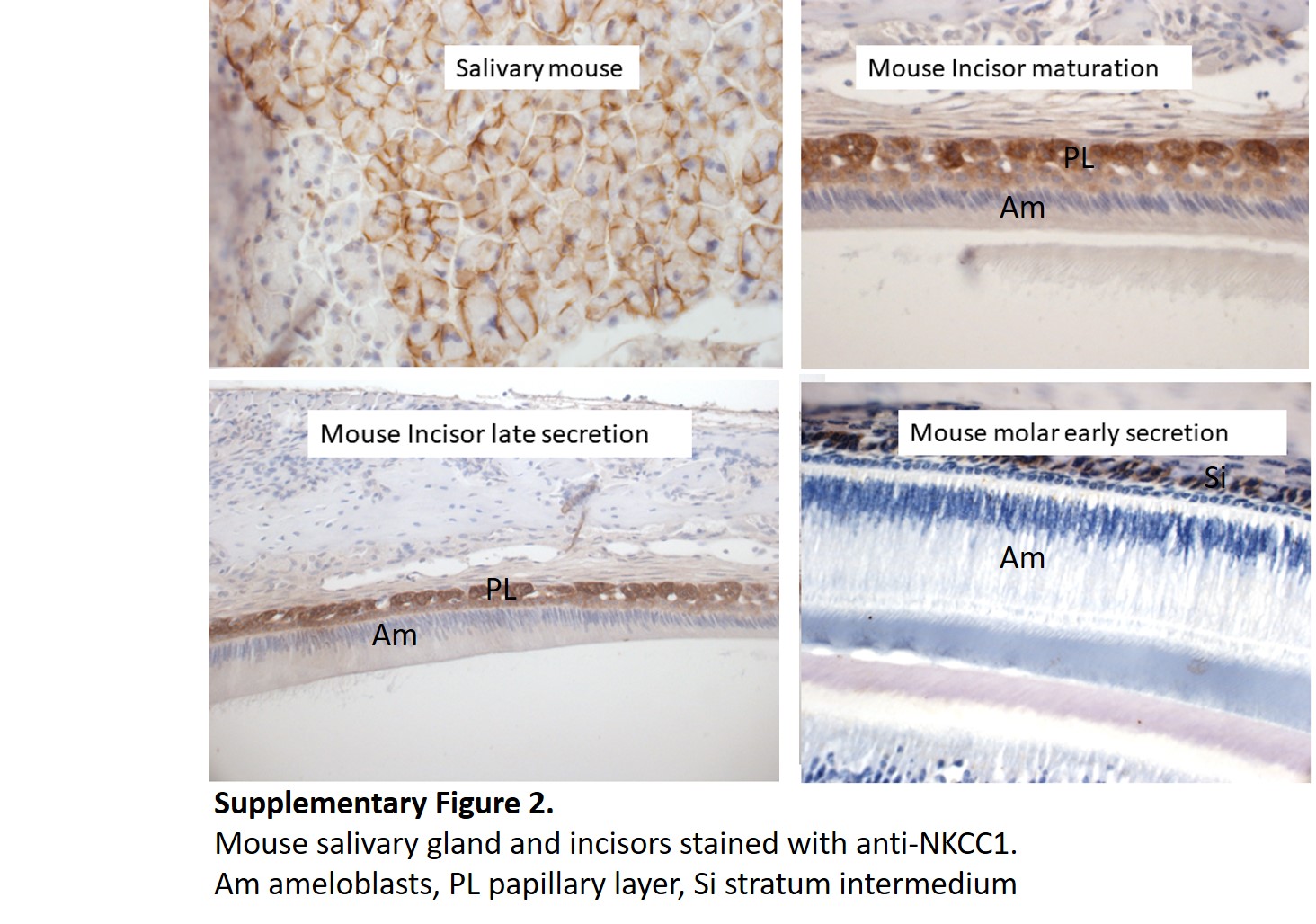

Supplement: Supplementary file 2 [file Image2.jpg]

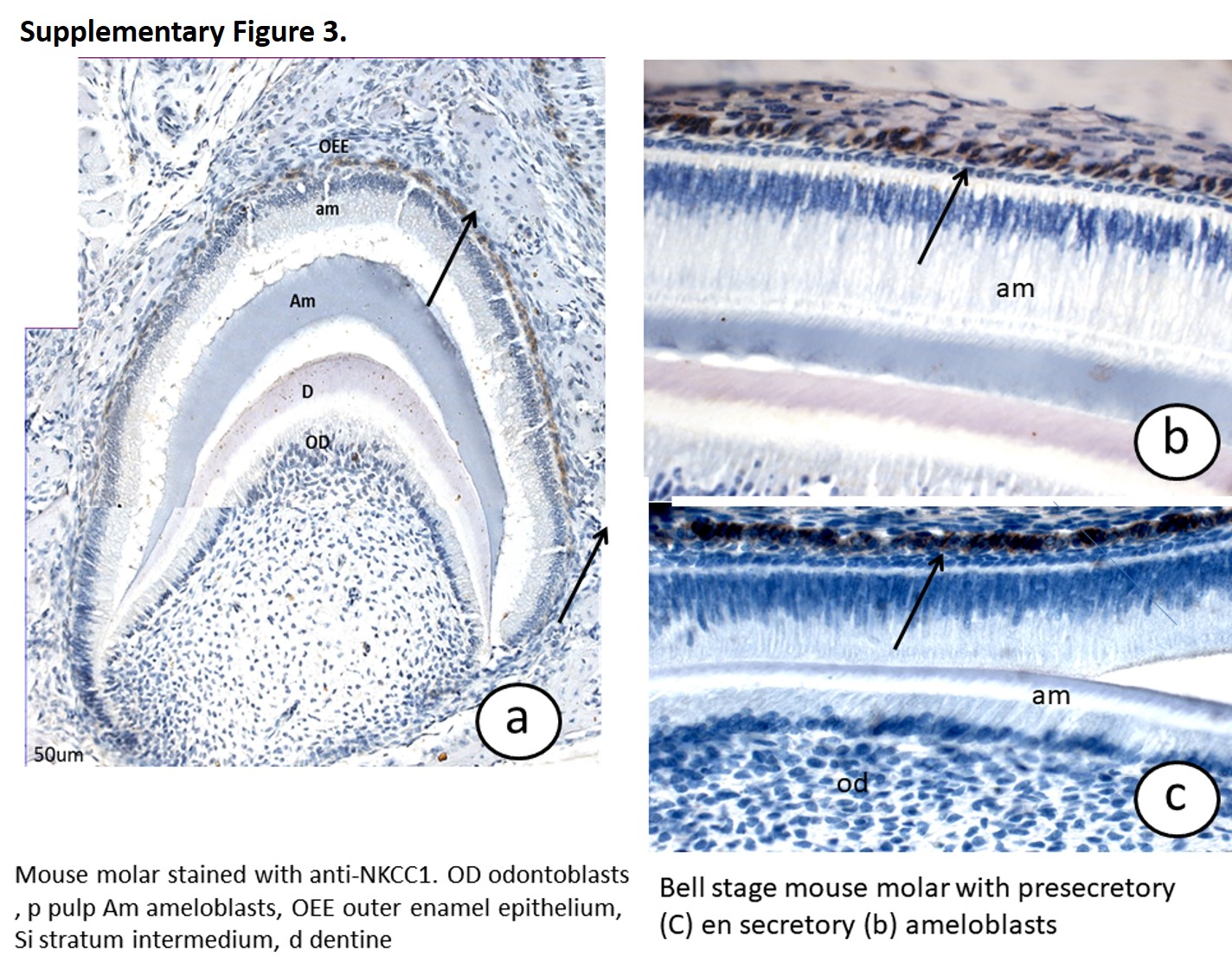

Supplement: Supplementary file 3 [file Image3.jpg]
